# Gender differences in myocardial function and arterio-ventricular coupling in response to maximal exercise in adolescent floor-ball players

**DOI:** 10.1186/2052-1847-6-24

**Published:** 2014-06-24

**Authors:** Åsa Fomin, Cristina Da Silva, Mattias Ahlstrand, Anders Sahlén, Lars Lund, Marcus Stahlberg, Anders Gabrielsen, Aristomenis Manouras

**Affiliations:** 1 Department of Medicine, Unit of Cardiology, Karolinska University Hospital, Stockholm, Sweden; 2 Department of Cardiology, Karolinska University Hospital, Stockholm, Sweden; 3 School of Technology and Health, Royal Institute of Technology, Stockholm, Sweden; 4 Department of Clinical Physiology and Cardiology, Karolinska University Hospital Huddinge, SE-141 86 Stockholm, Sweden

**Keywords:** Exercise, Echocardiography, Elastance, Tissue Doppler Imaging, Adolescent, Sex, Exercise stress test, Contractility, Peak VO2

## Abstract

**Background:**

The hemodynamic and cardiac responses to exercise have been widely investigated in adults. However, little is known regarding myocardial performance in response to a short bout of maximal exercise in adolescents. We therefore sought to study alterations in myocardial function and investigate sex-influences in young athletes after maximal cardiopulmonary testing.

**Methods:**

51 adolescent (13-19 years old) floor-ball players (24 females) were recruited. All subjects underwent a maximal exercise test to determine maximal oxygen uptake (VO2max) and cardiac output. Cardiac performance was investigated using conventional and tissue velocity imaging, as well as 2D strain echocardiography before and 30 minutes following exercise. Arterio-ventricular coupling was evaluated by means of single beat ventricular elastance and arterial elastance.

**Results:**

Compared to baseline the early diastolic myocardial velocity (E′LV) at the basal left ventricular (LV) segments declined significantly (females: E′LV: 14.7 +/- 2.6 to 13.6 +/- 2.9 cm/s; males: 15.2 +/- 2.2 to 13.9 +/- 2.3 cm/s, p < 0.001 for both). Similarly, 2D strain decreased significantly following exercise (2D strain LV: from 21.5 +/- 2.4 to 20.2 +/- 2.7% in females, and from 20 +/- 1 to 17.9 +/- 1.5% in males, p < 0.05 for both). However, there were no significant changes in LV contractility estimated by elastance in either sex following exercise (p > 0.05). Arterial elastance) Ea) at baseline was identified as the only predictor of VO2max in males (r = 0.76, p < 0.001) but not in females (p > 0.05).

**Conclusions:**

The present study demonstrates that vigorous exercise of short duration results in a significant decrease of longitudinal myocardial motion in both sexes. However, in view of unaltered end systolic LV elastance (Ees), these reductions most probably reflect changes in the loading conditions and not an attenuation of myocardial function per se. Importantly, we show that arterial load at rest acts as a strong predictor of VO2max in males but not in female subjects.

## Background

Numerous studies have documented the beneficial effects of moderate physical activity on cardiovascular health [1-3]. However, there is a substantial body of evidence supporting the notion that prolonged exercise might be less favorable regarding its immediate effects on cardiac function. Indeed, it has been demonstrated that ventricular performance is attenuated following strenuous exercise, a phenomenon that has been designated “exercise-induced cardiac fatigue” (EICF) [4]. Conversely, there is a paucity of data on EICF after a short bout of maximal exercise and the evidence available indicates that ventricular function is either unaltered or even improved during recovery [5]. While the physiological explanation of EICF is as yet not clear, some investigators support the notion that the observed alterations in cardiac function may be the result of preload reduction following exercise [6], whereas others advocate that a reduction of the intrinsic myocardial function might also contribute to the development of EICF.

Echocardiography is the method most frequently employed in studies of EICF due to its non-invasive nature and its broad availability. Moreover, the rapid development of instrumentation during the last decades has significantly enriched the arsenal of echocardiographic cardiac assessment with modalities such as tissue Doppler imaging (TDI) and 2D strain contributing substantially in the diagnostics of myocardial function. Although, the aforementioned echocardiographic modalities have been shown to provide robust quantification of the heart function, it is established today, that these techniques are susceptible to changes in hemodynamic loading. Conversely, ventricular elastance (E_es_) has been shown to be less load-dependent and the development of non-invasive algorithms for measuring E_es_ has provided novel possibilities for a more precise and robust quantification of the cardiac function.

To date, the studies investigating the EICF have mainly interrogated the cardiac alterations either in children or in adult population and a number of investigations have demonstrated significant sexual dimorphism in metabolic responses during exercise [7]. Despite the increasing number of teenagers participating in competitive sports during past decades [8,9], there is a lack of studies regarding the hemodynamic responses following short bouts of exercise in adolescent athletes. Furthermore, although significant sex-related differences after acute bout of exercise have been demonstrated [7,10], detailed knowledge is limited regarding this issue in adolescent exercise physiology. The lack of knowledge regarding EICF in adolescents impedes ones interpretation of the cardiovascular and respiratory responses to exercise and limits the guidance available for adolescents in competitive in exercise programs.

In light of the aforementioned and based on previously demonstrated sexual disparities in neuroendocrine responses during exercise we hypothesized that adolescents may behave in a different hemodynamic manner following a short bout of exercise. Thus, we undertook the present study on adolescent floor-ball players to investigate: 1. the myocardial and hemodynamic responses following maximal exercise testing and to assess whether intense exercise of short duration impacts on the myocardial function; 2. possible differences in heart function following exercise between the two sexes.

## Methods

### Study population

51 adolescent floor-ball players (24 female and 27 male), aged between 13 and 19 years old were recruited from a local floor-ball club. Floor-ball is a well known sport with its origin in Sweden and a significant number of participants of both genders. It is classified as a sport activity with a high aerobic and a low to moderate anaerobic component [11]. Informed consent was given from the participants and their parents following an oral and written explanation of the study objectives and protocol. The subjects reported initially to the study room and underwent a health and a physical examination. All subjects were healthy, had no history of cardiovascular disease and were not taking any medication. The investigation was approved by the ethical committee of Northern Stockholm (Young athlete Heart study, 2009/1246-31/1).

### Study protocol

#### Blood samples

Blood samples were collected from an indwelling catheter placed in the ante-cubital vein. Total hemoglobin, creatinine and N-terminal pro B-type natriuretic peptide (NT-proBNP) concentration were analyzed. Baseline venous blood gas samples were obtained (ABL 700, Radiometer, Copenhagen, Denmark) for analyses of blood lactate concentration and for metabolic assessment (base-excess, HCO_3_^-^, pH). Ten minutes post exercise, new blood samples were collected and analyzed.

#### Blood pressure recording

For the purposes of the echocardiographic measurements, systolic (SBP) and diastolic (DBP) blood pressure were recorded using a semi-automatic blood pressure (BP) measure device (Omron 7051 T, Omron Healthcare) directly after the termination of each echocardiographic examination with the patient supine. Additional BP recordings were obtained during the exercise test using a BP cuff and a Doppler flow measuring probe positioned over the radial artery.

#### Exercise test

A baseline supine resting 12-lead ECG was recorded and the subjects were subsequently placed on the exercise bicycle. One female participant was diagnosed with a Wolf-Parkinson-White type pre-excitation but was included in the study as she was completely asymptomatic. Following the study she was referred for cardiac electrophysiological evaluation. Her other exams and test results, beside the ECG, were completely normal. The exercise test was performed on a Rodby Ergometer Bike RE990 stress bicycle using a standard protocol (60 watt initial load applying load increments of 20 watt per minute). A 12 lead ECG was recorded at rest and during the whole exercise period.

#### Ventilation, gas exchange, pulmonary blood flow and cardiac output acquisition

During the exercise test, ventilation, O_2_ and CO_2_ were measured on a breath-by-breath basis using an Innocor® device (Innovision, Odense, Denmark) connected through a breathing mouth piece to the subject with a continuous inspiratory and expiratory sampling for the flow analysis. VO_2_max was derived as the average VO_2_ obtained during the last 30 seconds of exercise and normalized to the total body weight. Using the inert gas re-breathing technique, the Innocor® device provided measurements of the pulmonary flow at baseline and at peak exercise with the patient seated on the bicycle ergometer. To measure the pulmonary blood flow the subject performed a 30 seconds of closed re-breathing circle from a bag containing a gas mixture of 50% oxygen (O_2_), 0,5% Nitric Oxide (N_2_O) (soluble gas) and 0,1% sulphur hexafluoride (SF_6_) (insoluble gas) in nitrogen (N_2_) diluted with ambient room air. The re-breathing bag volume was set 30% higher than the expected tidal volume in each case. Pulmonary blood flow was calculated using standard validated formulas incorporated in the Innocor® software [12,13]. Pulmonary blood flow was assumed to equal cardiac output as there were no signs of pulmonary blood shunting. Subsequently cardiac index (CI) was calculated according to the standard formula.

#### Echocardiography

All subjects underwent transthoracic echocardiography (TTE) at two occasions i.e., prior the exercise and 30 minutes following the exercise termination, using a commercially available equipment (Vivid *i*, GE Vingmed, Horten, Norway) equipped with a standard phased array 2.5 MHz multi-frequency transducer. TTE was performed according to the current recommendations of the European Society of echocardiography [14] with the patient lying at the left lateral recumbent position. All measurements were performed offline by the same investigator (CDS) using commercially dedicated software (EchoPAC PC, version 11.0.0.0 GE Ultrasound, Waukesha, Wisconsin). Measurements were performed in three cardiac cycles and the values obtained averaged.

Measurements of the left ventricular (LV) end-diastolic diameter (LVEDD), septal and posterior wall thickness were assessed from the parasternal long-axis view using 2D. The LV mass was obtained according to the formula proposed by the American society of echocardiography [15]. From the apical window the LV end-diastolic (LVEDV), end-systolic (LVESV) volumes and ejection fraction (EF) were assessed employing the Simpson’s formula in 4- and 2 chamber apical view while ensuring that any apical foreshortening was avoided. Left atrial area (LA-area) was measured from the apical 4-chamber view. All volumetric measurements were indexed to Body Surface Area (BSA). BSA was calculated using the DuBois formula: BSA = (W^0.425^ × H^0.725^) × 0.007184. Transmitral flow velocities were obtained from the apical 4-chamber view using pulsed Doppler with the 5 mm sampling volume placed just below the tips of the mitral leaflets. Peak flow velocity during the early diastolic filling phase (E wave) and during the atrial contraction (A wave) as well as the deceleration time (DT) and the isovolumetric relaxation time (IVRT) i.e. the time between aortic valve closure and mitral valve opening were measured. The right ventricular (RV) diameter was assessed at end-diastole from the apical 4-chamber view at basal level of the RV. All measurements were performed on a minimum of 3 cardiac cycles and mean values were recorded.

#### Spectral tissue Doppler

Spectral tissue Doppler (TD) velocities were obtained by placing a 5 mm sampling volume at the basal segment of the septal and lateral LV wall and at the basal lateral RV wall. Care was taken in order to keep the sample volume located in the ventricular myocardium throughout the heart cycle and the angle of incidence as parallel as possible to the longitudinal axis of myocardial motion. The manufacturers’ default settings were kept unaltered (0 dB transmit gain and 3 dB receive gain). Accordingly, peak systolic (S′_LV_ and S′_RV_) velocities as well as peak diastolic velocities during early diastole (E′_LV_ and E′_RV_) and atrial contraction (A′_LV_ and A′_RV_) were acquired and the mean values from the septal and lateral wall were obtained and analyzed.

Moreover, measurements of myocardial isovolumic relaxation time (IVRT′), isovolumetric contraction time (IVCT′) and ejection time (ET′) were obtained from the RV lateral basal wall and the myocardial performance index (MPI) was calculated according to the equation: [(IVRT′ + IVCT′)/ET′].

#### Myocardial deformation analysis

Myocardial deformation analysis was performed offline using the 2D strain software provided in the EchoPac software. The cardiac cycle providing the best visual assessment of the endocardial borders was selected and all the measurements performed by single operator. The endocardial border was outlined manually and adjusted to match the actual borders of the myocardium and the regions of interest were optimized so that their thickness represented the corresponding thickness of the LV segments. Using the 2D strain analysis application provided by the EchoPac, the LV was automatically subdivided in 18 segments and 2D longitudinal strain was analyzed for the LV from the 4-, 2- and 3-chamber views. Subsequently the global longitudinal 2D strain was obtained by averaging the segmental strains. Peak systolic longitudinal strain for LV and RV was calculated at the aortic valve closure. According to the recommendations the 2D images were recorded in order to provide a frame rate between 70-80 frames per second. RV 2D strain was measured by tracing the RV lateral wall. 2D strain was measured on images obtained at baseline and 30 minutes after the termination of the exercise test.

#### LV Elastance and Arterial Elastance measurements

Using echocardiography and Doppler derived flow variables, effective arterial elastance (E_a_) and LV end-systolic elastance (E_es_) were calculated at baseline and 30 min following maximal exercise. E_a_ constitutes a “lumped index” of LV afterload in the time-domain and is calculated as E_a_ = LVESP/SV where LV end-systolic pressure (LVESP) was estimated as LVESP = 0.9 × SBP [16]. Arterial stiffness was defined as the reciprocal of compliance and assumed to be linearly related to pressure [17] and calculated as Stiffness = (SBP-DBP)/SV. Based on a two-element Windkessel model of the circulation with negligible central venous pressure, total peripheral resistance (TPR) was calculated as TPR = [DBP + (SBP-DBP)/3]/CO. [18] The relative importance of the components of E_a_ was studied after adjusting TPR for heart rate (HR) by dividing by the cardiac period (T).

E_es_ is defined as the slope of the line connecting the ESPVR of a run of heart beats recorded during a load intervention. Given its relative load-insensitivity, E_es_ is considered as the reference standard for measurements of LV contractility. E_es_ was estimated using the single-beat approach developed by Chen and co-workers [19]. Importantly, this method does not assume that the volume axis intercept of ESPVR is the origin of the diagram (0;0) but that the line can be extrapolated to intersect the volume axis at the point (V_0;0_) [19].

Briefly, E_es_ was calculated as E_es(sb)_ = [DBP - (E_Nd(est)_ × LVESP)]/[SV × E_Nd(est)_] where E_Nd(est)_ represents group-averaged normalized E_es_ values obtained as a function of EF and the ratio between SBP and DBP as described by the equation:

$$\begin{array}{ll} E_{\mathrm{Nd}\left(\mathrm{est}\right)}= & 0.0275-0.165\times \mathrm{EF}+0.3656\times \left(\mathrm{DPB}/\mathrm{SBP}\right)\\ & +0.515\times E_{\mathrm{Nd}\left(\mathrm{avg}\right)}. \end{array}$$

In this equation, E_Nd(avg)_ is given by a seven-term polynomial function:

$$E_{\mathrm{Nd}\left(\mathrm{avg}\right)}=\sum_{i=0}a_{i}\times t_{\mathrm{Nd}}^{i}$$

where summation is performed for i = 0 to 7, using values for a_i_ of [0.35695; -7.2266; 74.249; -307.39; 684.54; -856.92; 571.95; -159.1] respectively. The value of t_Nd_ was determined by the ratio of the pre-ejection period (R-wave to flow-onset) to the total systolic period (R-wave to end-flow), with the time at onset and termination of flow defined noninvasively from the pulsed Doppler waveform in LVOT.

### Statistical analysis

All data are presented as mean ± SD and as a ratio for categorical data. Student’s *t*-test and independent *t*-test were used when suitable for comparison of paired and unpaired data respectively. Normality was analyzed using Kolmogorov-Smirnov test. The Mann-Whitney rank sum test was employed for comparison of continues variables with skewed distribution. Spearman’s rho and Pearson’s correlation coefficient r was used as appropriate to examine the degree of correlation among variables.

Multiple regression analysis was performed for testing possible independent predictors of VO_2_max. The statistical significance was set at a two tailed probability level of p < 0.05. All analyses were performed with SPSS for Windows, release 18.0 (SPSS Inc. Chicago, III).

## Results

### Baseline demographic, metabolic and cardiopulmonary characteristics

Demographic characteristics of the study population are provided in Table 1. As shown the participants of the two sexes were of similar age and body mass index (BMI). Not surprisingly, hemoglobin and creatinine levels were higher in males as was the SBP at rest when compared to the female participants (*p <* 0.05). However, NT pro-BNP and troponine levels did not differ significant between the subjects of the two sexes. Female subjects exhibited a lower SBP (119 ± 11 mmHg; mean ± SD) compared to male participants (130 ± 13 mmHg, p < 0.05), whereas DBP did not differ between sexes (Table 1).

**Table 1:** Patient characteristics of the study population

**Patient characteristics**			
**Parameters**	**Male *(27)***	**Female *(24)***	**p-value**
Age (yrs)	17.0 ± 1.3	16.5 ± 1.8	ns
Height (cm)	179 ± 6	168 ± 5	<0.001
Weight (Kg)	69.7 ± 9.1	61.3 ± 9.2	0.002
BMI (kg/m^2^)	21.7 ± 2.7	21.6 ± 2.8	ns
Training per week (hours)	8.8 ± 1.3	7.4 ± 1.6	0.002
SBP (mmHg)	130 ± 15	119 ± 7	0.03
DBP (mmHg)	70 ± 8	71 ± 9	ns
Creatinine (μmol/L)	78 ± 9	65 ± 10	<0.001
Hemoglobin (g/L)	147 ± 8	132 ± 6	<0.001
VO2max (mL/min/Kg)	49 ± 5	39 ± 5	<0.001
LV mass_index_ (g/m^2^)	88 ± 15	85 ± 18	ns

BMI denotes body mass index; SBP, systolic blood pressure; DBP, diastolic blood pressure; VO2max; maximal oxygen uptake during peak cardiopulmonary testing normalized to total body mass; LVmass_index_, left ventricular mass normaliserad to body surface area.

### Cardiopulmonary and metabolic responses to exercise

Maximum SBP during exercise was lower in female (171 ± 20 mmHg) than in male subjects (185 ± 17 mmHg, p < 0.05), whereas maximum HR was similar (females: 191 ± 9 bpm vs. males: 194 ± 7 bpm). CI at maximum exercise was lower in females (7.0 ± 1.0 L/min/m_2_) than in males (8.3 ± 1.4 L/min/m^2^, p < 0.05).

RQ at maximum exercise was similar (females: 1.04 ± 0.06 vs. males: 1.05 ± 0.05, p > 0.05). VO_2_max normalized to total body mass was lower in females (39.1 ± 5.1 mL/min/kg; 51.2 ± 6.8 mL/min/kg) compared to males (48.7 ± 5.5 mL/min/kg; p < 0.05).

### Echocardiographic findings at baseline

None of the participants showed any evidence of structural or functional heart abnormality. Baseline echocardiographic characteristics are presented in Table 2. Despite larger EDVi and SVi (p < 0.05) in male participants, no differences were observed when comparing the LVEF. The RV end-diastolic diameter did not differ significantly between the participants of the two sexes when indexed to BSA (*p >* 0.05). TVI and 2D strain measurements recorded at baseline are presented in Table 2. As expected both male and female participants demonstrated excellent longitudinal LV and RV function as reflected by the high longitudinal myocardial velocities with similar values between the subjects of the two sexes (*p >* 0.05). The transmitral Doppler flow velocities and the DT were similar between the participants of the two sexes as were the MPI of the RV (*p >* 0.05).

**Table 2:** Echocardiographic and hemodynamic measurements evaluated before and following exercise in female and male floor-ball players

**Parameters**	**Pre-exercise**		**Post-exercise**	
	**Male *(n)***	**Female *(n)***	**Male *(n)***	**Female *(n)***
HR (bmp)	71 ± 12^†^ (27)	77 ± 10^†^ (24)	78 ± 11 (27)	84 ± 11 (24)
Systolic BP (mmHg)	130 ± 15*^†^ (27)	119 ± 7^†^ (24)	122 ± 9* (27)	114 ± 7 (24)
Diastolic BP (mmHg)	70 ± 8^†^ (27)	71 ± 9^†^ (24)	68 ± 10 (27)	68 ± 7 (24)
LVEDD (mm)	51 ± 4*^†^ (27)	48 ± 3^†^ (24)	49 ± 2*(27)	46 ± 3 (24)
LVESD (mm)	34 ± 3^†^ (27)	34 ± 4 (24)	33 ± 2 (27)	33 ± 3 (24)
LVEDV (mL)	121 ± 17*^†^ (27)	92 ± 19^†^ (24)	110 ± 18* (27)	86 ± 18 (24)
LVEDVi(mL/m^2^)	65 ± 7*^†^ (27)	54 ± 9^†^ (24)	59 ± 8* (27)	50 ± 8 (24)
LVESV (mL)	49 ± 8^†^ (27)	37 ± 9^†^ (24)	48 ± 10 (27)	36 ± 11 (24)
SV (mL)	74 ± 11*^†^ (27)	58 ± 9^†^ (23)	64 ± 10* (27)	53 ± 9 (23)
SVi (mL/m^2^)	40 ± 5*^†^ (27)	34 ± 5^†^ (23)	34 ± 5* (27)	31 ± 4 (23)
EF (%)	59 ± 4^†^ (27)	60 ± 5^†^ (24)	56 ± 5** (27)	60 ± 6 (24)
LA area (cm^2^)	18 ± 3*^†^ (27)	16 ± 3 (24)	17 ± 4 (27)	15 ± 3 (24)
2D Strain _LV_	20 ± 1.5**^†^ (27)	21.5 ± 2.4^†^ (24)	17.9 ± 1.5* (27)	20.2 ± 2.7 (24)
S′_LV_ (cm/s)	9.0 ± 1.0 (25)	8.5 ± 1.1 (23)	8.7 ± 1.3 (25)	8.4 ± 1.1 (23)
E′_LV_ (cm/s)	15.2 ± 2.2^†^ (25)	14.7 ± 2.6^†^ (23)	13.9 ± 2.3 (25)	13.6 ± 2.9 (23)
A′_LV_ (cm/s)	6.9 ± 1.6 (25)	6.1 ± 1.4 (23)	6.3 ± 1.5 (25)	6.4 ± 1.7 (23)
IVRT′ (ms)	62 ± 13 (26)	62 ± 13 (23)	61 ± 15 (26)	66 ± 14 (23)
E (m/s)	0.9 ± 0.2^†^ (27)	1 ± 0.2^†^ (24)	0.8 ± 0.1 (27)	0.9 ± 0.2 (24)
A (m/s)	0.4 ± 0.1 (27)	0.5 ± 0.1 (24)	0.5 ± +0.8 (27)	0.5 ± 0.1 (24)
DT (ms)	220 ± 52^†^ (27)	203 ± 43^†^ (24)	193 ± 51 (27)	188 ± 42 (24)
E_es_ (mmHg/mL)	2.9 ± 0.5 (27)	3.1 ± 0.6 (20)	2.7 ± 0.6* (27)	3.5 ± 1 (19)
E_a_ (mmHg/mL)	1.61 ± 0.23*^†^ (27)	1.86 ± 0.31 (19)	1.74 ± 0.28 (27)	1.98 ± 0.39 (18)
E_a_i (mmHg/mL/m^2^)	3.0 ± 0.5*^†^ (27)	3.1 ± 0.4 (19)	3.3 ± 0.7 (27)	3.3 ± 0.5 (18)
E_a_/E_es_	0.59 ± 0.1 (27)	0.63 ± 0.12 (18)	0.7 ± 0.33 (27)	0.59 ± 0.16 (18)
Stiffness (mmHg/mL)	0.82 ± 0.17 (27)	0.82 ± 0.25 (19)	0.86 ± 0.21 (27)	0.88 ± 0.2 (18)
TPR/T (mmHg/mL)	1.07 ± 0.21 (27)	1.21 ± 0.30 (19)	1.06 ± 0.17 (27)	1.16 ± 0.32 (18)
PCWP (mmHg)	9.4 ± 1.7** (26)	10.3 ± 1.7 (23)	9.2 ± 1.9** (27)	10.1 ± 1.6 (24)
RVEDD _basal_ (mm)	37 ± 4** (27)	34 ± 4 (24)	37 ± 5* (27)	33 ± 3 (24)
RA area (cm^2^)	15 ± 3**^†^ (27)	13 ± 2^†^ (24)	14 ± 3** (27)	12 ± 2 (24)
2D Strain _RV_	29.6 ± 4.7^†^ (27)	32.1 ± 4.5^†^ (23)	27.9 ± 4.1 (27)	29.8 ± 3.2 (23)
S′_RV_ (cm/s)	14.4 ± 2.2 (27)	14.5 ± 2.0^†^ (22)	14.4 ± 2.0 (27)	13.5 ± 1.7 (22)
E′_RV_ (cm/s)	17.3 ± 2.0^†^ (27)	17.7 ± 3.3^†^ (22)	15.4 ± 2.9 (27)	16.0 ± 4.0 (22)
A′_RV_ (cm/s)	10.1 ± 2.9 (26)	10.5 ± 2.8 (22)	9.5 ± 2.5 (26)	10.3 ± 3.4 (22)
MPI_RV_	0.4 ± 0.2^†^ (27)	0.4 ± 0.1^†^ (24)	0.5 ± 0.1 (27)	0.5 ± 0.2 (24)

Values are expressed as mean ± SD. HR denotes heart rate; BP, blood pressure; LVEDD, left ventricular (LV) end-diastolic diameter; LVESD, LV end-systolic diameter; LVEDV; LV end-diastolic volume; LVEDVi, LVEDV indexed to body surface area; LVESV, LV end-systolic volume; SV, stroke volume; SVi, stroke volume indexed to body surface area; EF, ejection fraction; LA, left atrium; 2D strain_LV_, 2-dimensional global LV strain; S′_LV_ E′_LV_ A′_LV_, mean value of peak systolic, early and lateral diastolic longitudinal myocardial velocity respectively in basal LV septal and lateral wall; E, peak early transmitral flow velocity, A; peak transmitral flow velocity due to atrial contraction; DT; deceleration time; E_es_; end-systolic LV elastance; E_a_, arterial elastance; E_a_i; arterial elastance normalized to body surface area; TPR/T, ratio between the total peripheral vascular resistance and the heart rate; PCWP, pulmonary capillary wedge pressure; RVEDD, right ventricular (RV) end-diastolic diameter at basal region of the RV; 2D strain_RV_, 2-Dimensional strain at the lateral RV wall; RA, right atrium; S′_RV,_ E′_RV_ and A′_RV_, mean value of peak systolic, early and lateral diastolic longitudinal myocardial velocity respectively in lateral RV wall; MPI_RV_, RV myocardial performance index; *Denotes statistical significance of p < 0.01 between the sexes; **Denotes statistical significance of p < 0.05 between the sexes; ^†^Denotes statistical significance of p < 0.05 between baseline and post exercise for the same sex.

On the other hand, LV longitudinal myocardial 2D strain and RV 2D strain was higher in females (LV global 2D strain: males: -20 ± 1%; females: -21 ± 2%; RV 2D strain: males: -29 ± 4% vs. females: -32 ± 5%, p < 0.05 in both cases).

### Echocardiographic alterations in response to exercise

2D measurements obtained 30 minutes after the maximal cardiopulmonary test are shown in Table 2. Significant reductions in LVEDV and LVESV were observed in both sexes as compared to the baseline measurements.

Despite a significantly higher HR as compared to baseline, E′_LV_ was decreased following examination both at the septal and lateral wall (*p <* 0.05). Furthermore, male and female participants showed a reduction in global LV longitudinal 2D strain following exercise (*p <* 0.05). Early transmitral inflow velocities (E) were significantly lower compared to baseline measurements in both males and females. Similar to the observations for the LV, significant reduction in E′_RV_ and 2D strain_RV_ were observed after the exercise in both sexes with higher values obtained in female participants as compared to males (*p <* 0.05). Furthermore, subjects of both sexes revealed a significant increase of the RV MPI _index_ as a result of longer isovolumic myocardial phases and a shorter ejection period.

### Measurements of LV and Arterial Elastance at baseline and in response to exercise

At baseline, LV systolic performance as evaluated by single beat E_es_ measurements demonstrated similar values in male and female participants (p > 0.005) with similar arterial load as estimated by E_a_.

At peak exercise, CI was higher in male participants (8.3 ± 1.4 vs. 7.0 ± 1.0 L/min/m^2^, p < 0001) and taking into account that maximal HR did not differ between sexes (194 ± 7 vs. 191 ± 9 bpm, p > 0.05), the sole factor responsible for this difference was a higher SVi in males compared to females (43 ± 7 vs. 37 ± 6 mL/m^2^, p < 0001).

E_a_i increased significantly compared to baseline values with participants of the two sexes displaying similar values (male: 4.0 ± 0.7; females 4.3 ± 0.8 mmHg/mL, p > 0.05) despite significantly higher systolic BP in males (males: 185 ± 17 mmHg; females 171 ± 20 mmHg, p < 0.001).

**Figure 1:**
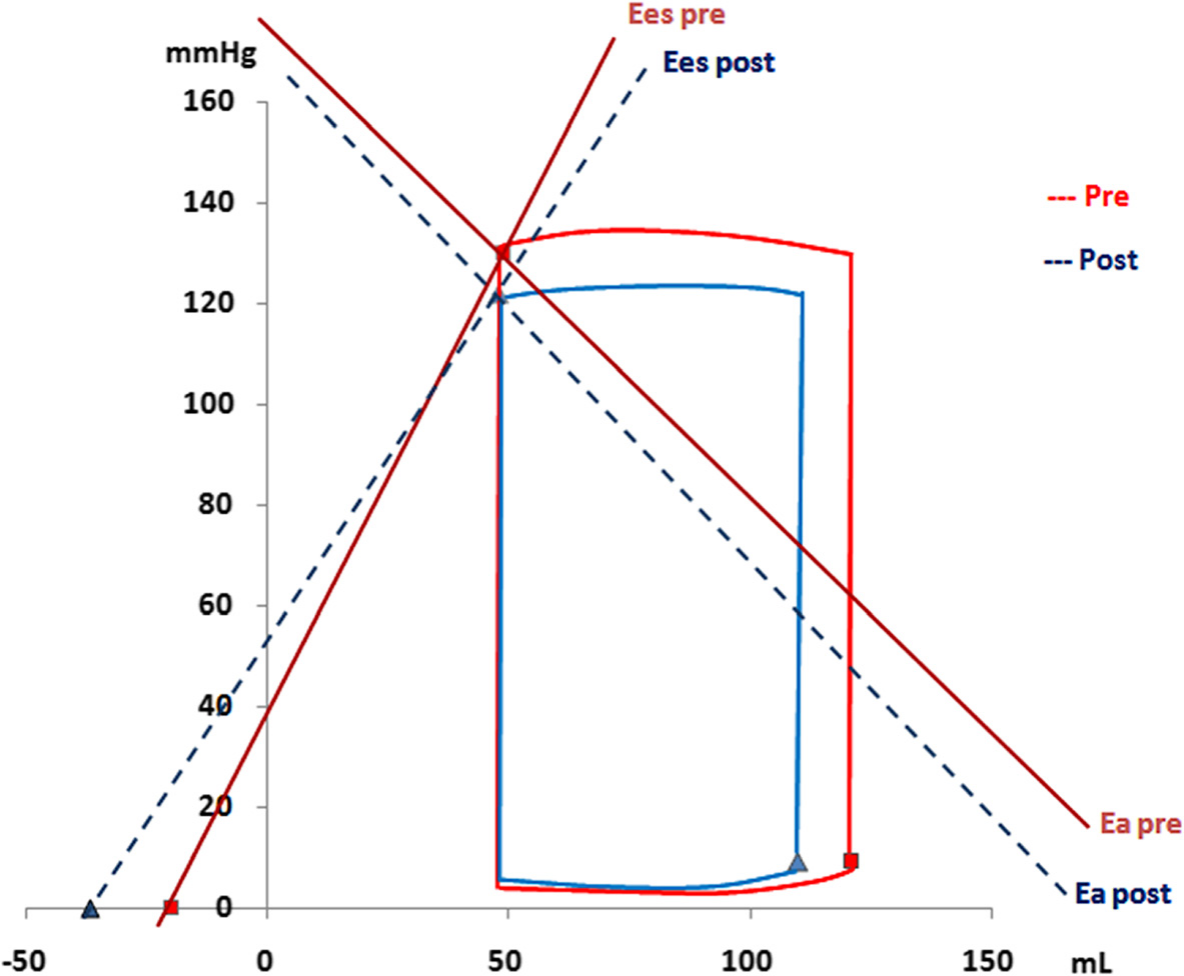
**The pressure-volume relationship in male floor-ball players at baseline and following maximal cardiopulmonary testing.** The slope of left ventricular (LV) end-systolic pressure and volume (ESPVR) relationship illustrates the LV contractility and was measured using single-beat LV elastance (E_es_). The arterial elastance (E_a_) was measured as the ratio between the LV end-systolic pressure and the stroke volume (SV). The figure illustrates the alterations in E_es_ and E_a_ prior (solid lines) and following the exercise (dashed lines).

**Figure 2:**
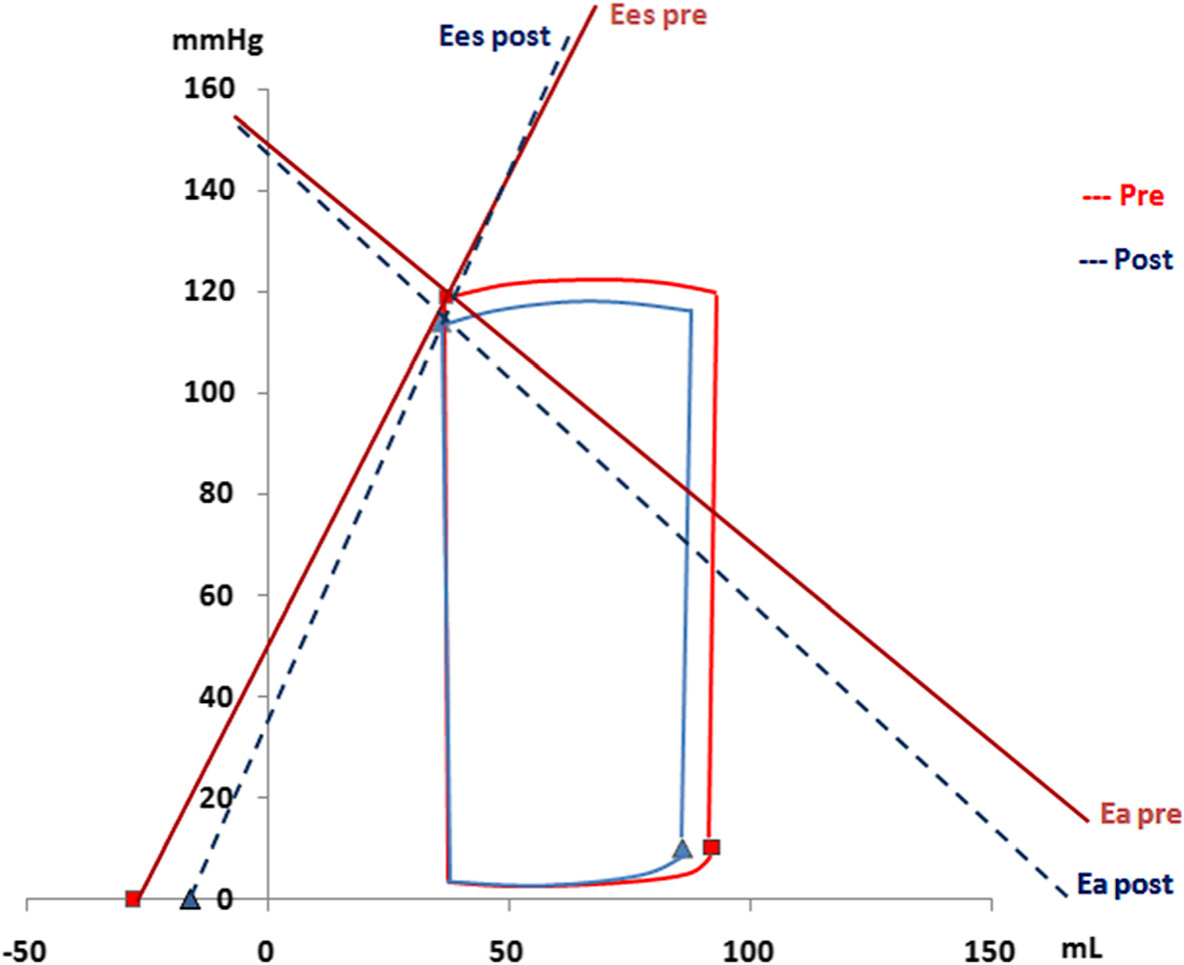
**The pressure-volume relationship in female floor-ball players at baseline and following maximal cardiopulmonary testing.** The slope of left ventricular (LV) end-systolic pressure and volume (ESPVR) relationship illustrates the LV contractility and was measured using single-beat LV elastance (E_es_). The arterial elastance (E_a_) was measured as the ratio between the LV end-systolic pressure and the stroke volume (SV). The figure illustrates the alterations in E_es_ and E_a_ prior (solid lines) and following the exercise (dashed lines).

Following exercise, no significant differences in E_es_, E_a_i or E_a_/E_es_ ratio were observed as compared to baseline values with higher E_es_ levels in female participants (p < 0.05, Table 2, Figures 1 and 2).

In order to investigate whether baseline LV and arterial elastance measurements correlated with baseline levels of NT-proBNP and troponine or changes in these measurements, regression analysis was performed failing to demonstrate a significant relation between the measurements.

**Figure 3:**
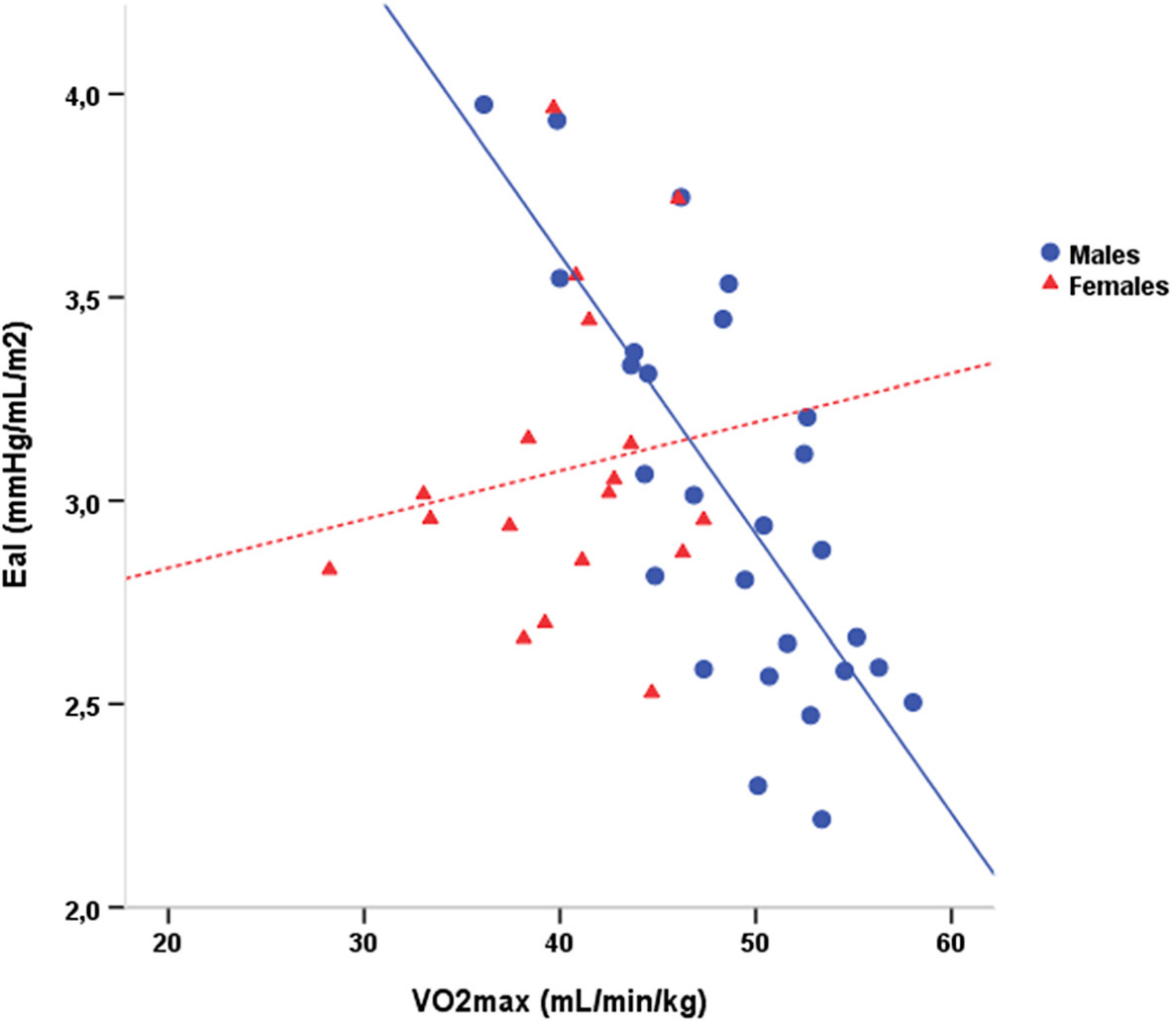
**The relationship between the baseline arterial elastance normalized to body surface area (E**_**a**_**i) and the maximal aerobic capacity as measured by VO2max normalized to the total body mass.** As illustrated there was a strong inverse correlation between the E_a_i and VO2max in male but not in female adolescents.

Correlation analysis revealed that for the entire group, VO_2_max normalized to the total body mass was significantly associated with resting EDVi (r = 0.3, p = 0.036) and inversely related with E_a_i (r = -0.36, p = 0.01), but not with E_es_ (p > 0.05) or E_a_/E_es_ ratio. However, when these correlations were tested for each sex separately the results differed. Specifically, in boys there was a strong inverse correlation between E_a_i and VO_2_max (r = -0.76, p < 0.001) (Figure 3). In the regression analysis performed including E_es_, E_a_i, E_a_/E_es_ and EDVi, E_a_i was identified as the only predictor of VO_2_max standing for 55.5% of the variability in VO_2_max. The regression equation wasVO_2_max = 73.7-8.34 × E_a_i, with an overall model fit of r = 0.76, F (1, 25) = 33, p < 0.001. When the analysis was limited only to participants reaching RQ ≥ 1.05 at peak exercise the correlation pattern became even stronger with the E_a_i in males being the only independent predictor standing for 65% of the explained variability in VO_2_max. The regression equation in that case was VO_2_max = 78.9-10.2 × E_a_i, with an overall model fit of r = 0.83, F (1, 13) = 27, p < 0.001. On the other hand, in females VO_2_max was not related with any of the tested variables (p > 0.05), (Figure 3).

### Diastolic LV function and myocardial stiffness in response to exercise

**Figure 4:**
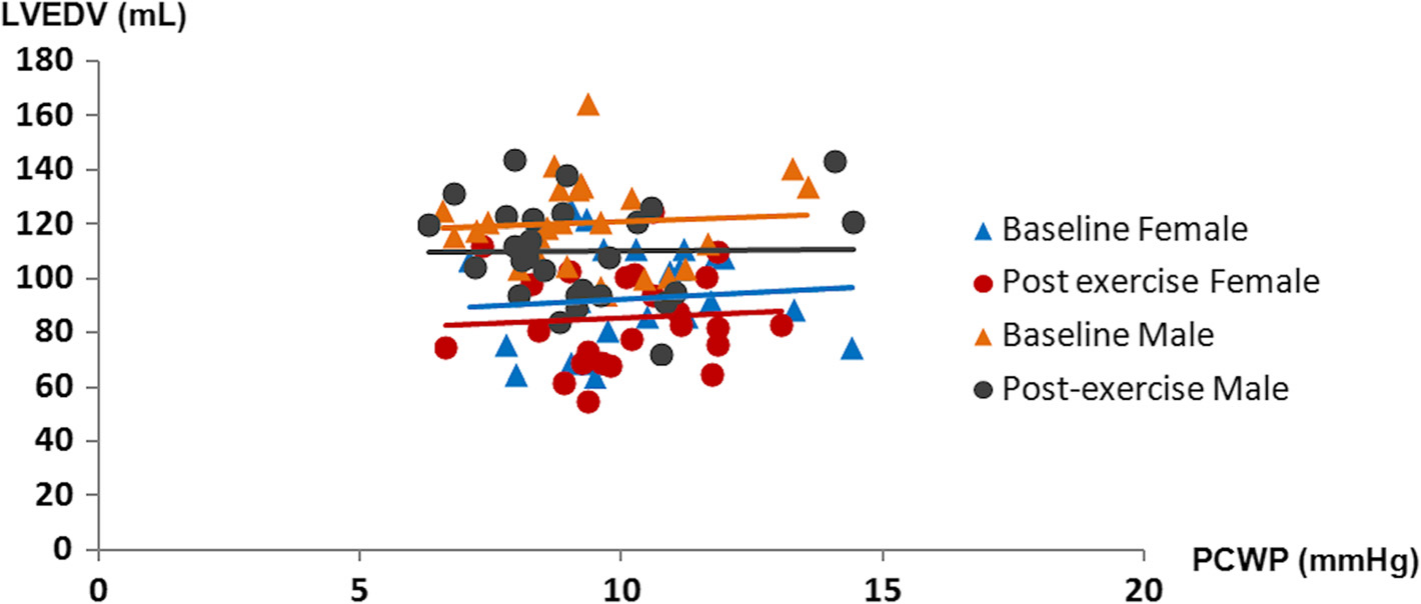
**The end-diastolic pressure volume relationship (EDPVR) in male and female participants before and after maximal cardiopulmonary testing.** The end-diastolic left ventricular (LV) pressure (LVEDP) was considered to equal the pulmonary wedge pressure and was estimated non-invasively by a previously described equation. LV end-diastolic stiffness illustrated by the EDPVR slope (solid lines) was similar for both sexes at baseline and was essentially unaltered following exercise.

Pulmonary capillary wedge pressure (PCWP) as estimated by the previously proposed equation [20] did not significantly change following the bout of maximal exercise testing in either sex. Similarly as shown in Figure 4, no changes in the slope of LVEDV to PCWP ratio were observed after exercise indicating unaltered LV end-diastolic stiffness.

## Discussion

In this investigation we show that despite a significant reduction in longitudinal ventricular function, myocardial contractility remains essentially unaltered following a bout of maximal exercise testing in adolescent floor-ball players of both sexes. Furthermore, we demonstrate that the arterial load and the end-diastolic LV dimension at rest act as the only independent predictors of aerobic capacity in male but not in female participants.

The cardiopulmonary and metabolic responses to a maximal exercise testing have been well studied in adults and differences between sexes in this setting have been previously described [21]. Recently, we reported the results regarding the aerobic cardiopulmonary responses between males and females in the present cohort. Briefly, male and female adolescent participants exhibited comparable increases in HR and similar metabolic adaptation as pH, HCO3 -, lactate levels and the respiratory ratio (RQ) [22]. Additionally in terms of VO_2_max, we showed that although females and males differ significantly when comparing values of VO_2_max indexed to total body mass no differences were observed when VO_2_max was normalized to estimated leg muscle mass [22].

### Ventricular systolic performance in response to exercise

The issue of cardiac function in response to exercise has been previously investigated and there is growing body of evidence indicating that prolonged exercise may yield reduction in LV longitudinal performance [23-25]. Conversely, the effects of short bout of rigorous exercise on myocardial function are less clear [26]. In our cohort both the early diastolic velocities (E′_LV_) and the longitudinal 2D strain declined following exercise which, taking into account that these two measurements constitute sensitive markers of the LV function, implies attenuated LV performance at recovery following exercise [27,28]. However, when LV function was further evaluated using the end-systolic pressure-volume relationship (ESPVR), a method robust to load changes, LV contractility as estimated by E_es_ remained essentially unaltered in participants of both sexes following exercise. Whereas E′_LV_ as well as 2D strain have been proposed as being relatively load independent [29,30], later reports have demonstrated the influence of load alterations on these measurements [31,32]. In this context, the current findings suggest that the reductions in longitudinal LV strain and E′_LV_ may not explicitly represent reduction of the inherent myocardial contractility but might rather reflect exercise-induced changes in hemodynamic loading. Scott and associates in their study on triathletes demonstrated significant gender differences following prolonged strenuous exercise, with male participants exhibiting attenuated chronotropic and inotropic response to β-adrenergic stimulation [7]. The authors suggested that this might be partly the result of increased sympathetic response in males during exercise and a subsequent desensitization of cardiac β-receptors [7]. This gender-based disparity in β-receptor responsiveness has been observed in studies employing prolonged exercise [7,33]. Although the effect of short-duration maximal exercise on adrenergic cardiac receptors has not been yet investigated, possible similar influence could partly explain our findings as we demonstrate significantly higher E_es_ values in females compared to males post-exercise.

Similar to previous studies, we show a significant reduction of LVEDV in participants of both sexes after maximal exercise testing [34,35] whereas E/E′_LV_, a well established surrogate of LA pressures, was essentially unaltered. These observations together with a significant reduction in E′_LV,_ a marker of early LV myocardial relaxation, advocate for a decrease in LV filling without a concomitant decrease in preload as expressed by LA pressures. Previous studies have suggested that the attenuation in LV filling following exercise could be the result of changes in myocardial relaxation and reduced suction effect during early diastole [36]. However, in our study, the lack of significant changes in myocardial relaxation as measured by IVRT′ does not support the notion of intrinsic exercise-induced alterations in early myocardial diastolic performance. On the other hand, there is growing evidence showing that RV function influences the LV mechanics and LV filling [36]. Employing TVI and 2D strain, it has been previously shown that following prolonged exercise, the longitudinal RV function is reduced [2]. Conversely, Poh and colleagues reported that strenuous exercise of short duration, resulted in augmented E′_RV_ as compared to baseline [26]. Opposed to this later study our findings show that both the RV strain and the early myocardial velocity of the RV free wall declined significantly following exercise, a discrepancy that might be the result of differences in study protocol. Indeed, in the present study the examination was performed 30 minutes after maximal cardiopulmonary testing and consequently the HR was similar to baseline whereas in the study of Poh et al. HR was significantly higher following exercise which has been shown to yield higher E′_LV_[37]. Furthermore, the essentially unaltered RV dimensions together with a concomitant reduction in RV performance as measured by RV strain and E′_RV_, suggests that the reduced LV filling might be the result of a reduction in the RV stroke volume rather than the consequence of a mere decrease in LV preload. In addition, as the two ventricles share a common septal wall, alterations in the RV function might influence LV diastolic function, impairing thus LV filling [36].

### Aerobic capacity and hemodynamics

In line with previous reports, we demonstrate that male participants as compared to girls exhibited higher CI and aerobic capacity as measured by VO_2_max adjusted to total body mass. The differences in aerobic response between the two sexes are discussed in detail in our previous report [22]. It have shown that the response of arterial load may vary during exercise, showing increase, decline or even remaining unaltered at peak exercise. In our study, only four participants (three females) demonstrated E_a_ reduction (1-10% decrease from baseline values) while the rest showed significant E_a_ increment (median increase 30%, range 3 to 131%). In a recent investigation on healthy adults no significant influence of age and sex on *Δ*E_a_i was found [38]. In line with this observation, we show that male and female participants behave in a similar pattern regarding E_a_i changes at peak exercise. In our cohort, *Δ*E_a_i was inversely related with baseline E_a_i and this relationship was very similar in the two sexes (*Δ*E_a_i vs. E_a_i rest; females: r = -0.5, p = 0.003, males r = -0.51, r = 0.008) which is in accordance with previous observations in healthy adults [38]. This implies that a more compliant arterial system at rest may shift to lesser degrees of compliance during exercise. Unfortunately the design of this study does not allow us to provide an answer to possible mechanisms that may contribute to this phenomenon.

There is increasing recognition on the importance of the arterio-ventricular interaction and the arterial load on the cardiac performance. Fahs et al. have recently described that the coupling ratio of arterial to ventricular elastance (E_a_/E_es_) is inversely related to the aerobic capacity as described by VO_2_max [39] in female but not in male young endurance athletes. Based on this observation, we investigated whether baseline hemodynamic and echocardiographic variables could act as predictors of the aerobic capacity. In a model that included E_a_, E_es_, E_a_/E_es_ as well as EDVi, we found that in the whole study group, E_a_i at baseline significantly correlated with aerobic capacity and acted as the single independent predictor of VO_2_max (beta = -0.41, model fit = 0.41, p < 0.005). However, it appears that sex influences this relationship as E_a_i was a strong and the only predictor of the aerobic capacity in male participants (beta = -0.76, model fit = 0.76, p < 0.001) but lacked any significant correlation with VO_2_max in females. When the analysis was limited to participants that exhibited RQ ≥1.05 at peak effort the correlation between E_a_i and VO_2_max became even stronger in males. Additionally, in male participants the degree of correlation between E_a_i and VO_2_max persisted even when VO_2_max was adjusted for muscle leg mass, fat-free mass and trunk muscle mass (r = -0.7; r = -0.74, r = 0.74, p < 0.001 respectively) while no correlation was observed in females. Fahs and coauthors showed a weak inverse asssociation between E_a_ and VO_2_max in their whole study cohort, but when sexes where separately examined no significant relationship was found between these parameters [39]. Similar to our findings, E_es_ failed to demonstrate significant association with aerobic capacity in that study [39]. The physiologic explanation for this discrepancy between sexes is unclear as the main determinants of E_a_i i.e. the arterial stiffness, the TPR and the HR were similar in males and females prior and following exercise. Possible differential responses of the arterial system during exercise cannot be ruled out, as the design of the current study does not allow further interrogation regarding this issue. Previous investigations have indicated that in women there is mainly a parasympathetic regulation of the cardiovascular system at rest whereas in males the sympathetic activity is dominating both at rest [40] and during exercise [41], a disparity that may stand for the currently demonstrated discrepancy between sexes regarding the association of E_a_ and VO_2_max in our study. A possible conditioning of the cardiovascular system during exercise might also contribute to the disparate relationship between E_a_i and VO_2_max in the two sexes as the frequency of training between sexes was significantly different with boys reporting more hours of training (9 ± 1 h/week) compared to females (7 ± 2 h/week).

## Limitations

The present study was performed in adolescent floor ball athletes with an average of roughly 7 hours of physical exercise per week implying that the current results do not pertain to elite athletes. Additionally, although the participants of the two genders were matched according to age, we did not interrogate sexual maturation indices in our cohort which might constitute a confounder in the present study. A considerable part of study results were obtained using Doppler based methods. This implies that the results are subjected to possible suboptimal insonation angle. However, the impact of this possible influence is not considered to be significant as care was taken for the angle of incidence not to exceed 20 degrees. Measurements of cardiac output using inert gas are influenced in the presence of significant pulmonary or intracardiac shunting. The TTE examination did not reveal any signs of atrial or ventricular septal defect. However, we did not perform contrast echocardiography prior or during the exercise and thus the possibility of pulmonary shunting cannot be excluded and thus possible influence on the results of our study especially for cardiac output measurements during exercise cannot be fully excluded. Furthermore measurements of LV elastance were performed using the single-beat approach rather than conductance catheters which is considered the reference standard in this setting. However, the single beat method has been well validated against conductance catheters and provides reproducible results [19].

## Conclusion

In conclusion, we demonstrate that LV myocardial responses to maximal cycle exercise did not differ between the female and male participants. Furthermore, we conclude that exercise induced reduction in ventricular function assessed by quantitative and Doppler measurements although comparable in both sexes may not indicate a decrease in intrinsic myocardial contractility. Additionally we show that arterial load at rest is a strong predictor of aerobic capacity in male but not female adolescent floor-ball players. Further studies are needed to investigate the differential effect of arterial load between the two sexes on maximal aerobic performance.

## Competing interests

The authors declare that they have no competing interests.

## Authors’ contributions

ÅF has made substantial contribution for the data acquisition (information and subject recruitment, supervised and recorded data during exercise stress testing and blood sampling) and for the conception and design of the study. Has also been involved in the drafting of the manuscript as well as given final approval of the final version of the manuscript. CDS has made substantial contribution for data analysis (echocardiographic data analysis off-line) and interpretation (statistics); conception and design of the study. Has also been involved in the drafting and critical revision of the manuscript as well as given final approval of the final version of the manuscript. MÅ has made substantial contribution for the data acquisition (information and subject recruitment, supervised and recorded data during exercise stress testing and blood sampling) and for the conception and design of the study. Has also been involved in the drafting of the manuscript as well as given final approval of the final version of the manuscript. AS has made substantial contribution for data interpretation the conception and study design. Has also been involved in the drafting and critical revision of the manuscript as well as given final approval of the final version of the manuscript. LL has made substantial contribution for data interpretation; conception and design of the study. Has also been involved in the drafting and critical revision of the manuscript as well as given final approval of the final version of the manuscript. MS has made substantial contribution for the data acquisition and for the conception and design of the study. Has also been involved in the drafting of the manuscript as well as given final approval of the final version of the manuscript. AG has made substantial contribution for data acquisition (information and subject recruitment, supervised and recorded data during exercise stress testing and blood sampling, Innocor examination), analysis and interpretation; conception and design of the study. Has also been involved in the drafting and critical revision of the manuscript as well as given final approval of the final version of the manuscript. AM has made substantial contribution for data acquisition (echocardiographic examination and off-line analysis and interpretation (statistical analysis); conception and design of the study. Has also been involved in the drafting and critical revision of the manuscript as well as given final approval of the final version of the manuscript. All authors read and approved the final manuscript.

## Authors’ information

Åsa Fomin and Cristina DaSilva were equal first author contributors. Aristomenis Manouras and Anders Gabrielsen were equal senior author contributors.

## Pre-publication history

The pre-publication history for this paper can be accessed here:

http://www.biomedcentral.com/2052-1847/6/24/prepub
